# Industrial Production of Functional Foods for Human Health and Sustainability

**DOI:** 10.3390/foods13223546

**Published:** 2024-11-06

**Authors:** Xinrui Yuan, Moyu Zhong, Xinxin Huang, Zahid Hussain, Maozhi Ren, Xiulan Xie

**Affiliations:** 1 Functional Plant Cultivation and Application Teams, Institute of Urban Agriculture, Chinese Academy of Agricultural Sciences, Chengdu 610000, China; 2 School of Agricultural Sciences, Zhengzhou University, Zhengzhou 450052, China

**Keywords:** functional agriculture, functional food, nutrient fortification, bioactive ingredients, industrial production

## Abstract

Functional foods significantly affect social stability, human health, and food security. Plants and microorganisms are high-quality chassis for the bioactive ingredients in functional foods. Characterised by precise nutrition and the provision of both nutritive and medicinal value, functional foods serve a as key extension of functional agriculture and offer assurance of food availability for future space exploration efforts. This review summarises the main bioactive ingredients in functional foods and their functions, describes the strategies used for the nutritional fortification and industrial production of functional foods, and provides insights into the challenges and future developments in the applications of plants and microorganisms in functional foods. Our review aims to provide a theoretical basis for the development of functional foods, ensure the successful production of new products, and support the U.N. Sustainable Development Goals, including no poverty, zero hunger, and good health and well-being.

## 1. Introduction

Earth faces numerous threats, including increased human activity and global climate change. The escalation of climate change and global warming, sharp decline in biodiversity, severe scarcity of water resources, environmental pollution and ecotoxicity, depletion of resources, impending threat of nuclear war, and tensions arising from the arms race are among these challenges. The consequences of failing to address these challenges are severe and affect human health, food security, and the stability of the natural environment.

Advancements in traditional breeding techniques, modern biotechnology, and innovations in agronomic practices have contributed significantly to the establishment of sustainable food systems [1,2]. There are many ways to build a sustainable food system, one of which is by-product recycling, such as extracting active substances from the processing by-products of vegetables and fruits and employing these useful compounds from such waste in value-added products. Replacing animal-based products with non-animal products, such as the now popular microalgae single-cell protein, is also a way to reduce CO_2_ emissions and protect ecology. In addition, the application of plant factories significantly alleviates stress on arable land and water resources; cell factories replace chemical processing by using clean biological processing methods that are environmentally friendly, efficient, and sustainable [3,4].

However, despite these advances, one-third of the global population remains malnourished, and more than two billion people suffer from micronutrient deficiencies [5]. This has sparked a strong interest in healthy eating and led to a gradual shift in dietary concepts from mere satisfaction to the desire for high-quality, healthy diets (i.e., simultaneously eating well and healthily) [6]. Advances in biotechnology have significantly contributed to this process by integrating the health and agricultural sectors. Scientific and technological advancements have enabled the constant revelation of the complex relationships between food composition, dietary structure, nutritional conditions, and human health, thus creating the possibility of enhancing physical fitness and immunity through diet [5,7,8]. With the application of modern biotechnology, cultivation of plants and microorganisms enriched with specific nutrients or bioactive ingredients has become a reality [9,10]. Therefore, the bioeconomy offers technical solutions and promotes strategies to mitigate some of the biggest global challenges, which are crucially important for the safeguarding of human life and health.

Functional foods are defined as foods that contain biologically active compounds which, in defined, effective, and non-toxic amounts, provide a clinically proven and documented health benefit by utilising specific biomarkers for the prevention, management, or treatment of chronic diseases or their symptoms. Characterised by precise nutrition and the provision of both nutritive and medicinal value, functional foods serve as key extension of functional agriculture. Functional agriculture refers to the production of functional products through biofortification or other biotechnological strategies, thereby providing raw materials with adequate nutrients for functional foods. Functional foods provide an optimal solution to the global issues of malnutrition and hidden hunger [11]. However, functional foods should not be mistaken for medicines, as a regulatory framework controls the authorisation of health claims. According to the current European regulation (EC 1924/2006), any health claim should be supported by evidence from human interventions, such as clinical trials. Health claims approved by governmental health agencies (e.g., the European Food Safety Authority, EFSA) are perceived as trustworthy by consumers [12].

**Figure 1:**
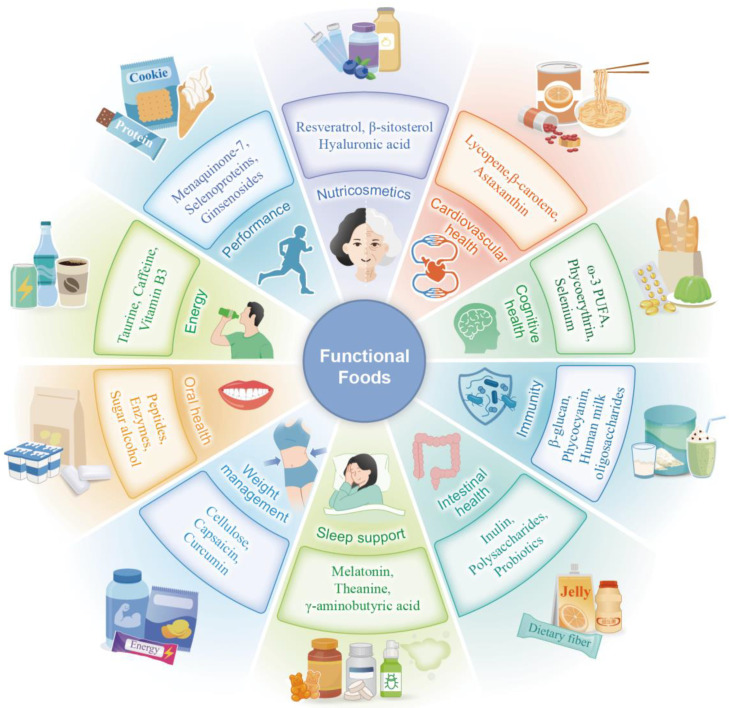
Types of functional foods, bioactive ingredients, and common products. This schematic of concentric circles shows the title in the innermost circle followed by a circle containing the current concepts, reflecting 10 condition-specific broad categories of functional foods. The penultimate circle illustrates the primary active components used to create the aforementioned ten types of functional foods. Typical examples of well-linked functional foods in each category are shown in the outermost circle. PUFA: polyunsaturated fatty acids.

Functional foods comprise various categories, including traditional foods, fortified foods, and dietary supplements. Their functional properties can be further categorised into energy supplementation, performance enhancement, skin nourishment, cardiovascular disease prevention, cognitive health promotion, immune system enhancement, weight management, and oral health maintenance (Figure 1). Recently, there has been a substantial increase in research on the applications of functional foods. Over the past five years, the term “functional food” has been included in 32,143 publications according to statistics from the Web of Science database [13]. According to data published by Grand View Research, the market for functional foods has increased continuously and is expected to increase to USD 586.069 billion by 2030 [14].

Reviews of the relevant literature have primarily focused on the historical development of the definition of functional foods, functional food processing techniques [15], approaches to functional food product development [11], and the significance of functional foods for human health [16]. Here, we systematically summarise the bioactive ingredients of functional foods and their specific functions, analyse strategies for nutritional fortification and industrial production of functional foods, and provide insights into the challenges and prospects for the application of plants and microorganisms in functional foods. Consistent with the 17 Sustainable Development Goals, which provide a shared blueprint for peace and prosperity for people and the planet, this review aims to provide valuable insights and practical guidance for the development of functional foods and ensure the successful production of new products, thereby contributing to the sustainable development of the functional food industry to achieve no poverty, zero hunger, and good health and well-being.

## 2. Bioactive Ingredients in Functional Foods and Their Effective Functions

The physiological benefits of functional foods stem from the bioactive ingredients and functional factors contained in their raw materials, which primarily include amino acids, peptides, proteins, functional polysaccharides, polyunsaturated fatty acids, vitamins and their analogues, mineral elements, trace compounds, and dietary fibre. These functional components are essential for maintaining and improving human health, and the specific effects of each key component are discussed in detail in the following sections.

### 2.1. Amino Acids, Peptides, and Proteins

Proteins account for 45% of the total dry matter in the body and 70% of the total muscle mass. They are closely related to metabolism, anti-disease and antibacterial immunity, fluid balance, and the transmission of genetic information in the body [17]. Proteins can be grouped according to their amino acids (essential, semi-essential, or non-essential). Based on their source, proteins can also be categorised as animal, plant, or microbial proteins. Plant and microbial proteins have gradually gained popularity because of various factors, such as sustainability, dietary diversity, animal welfare, and health benefits associated with non-animal proteins. These proteins have the potential to replace conventional animal proteins, such as eggs, lean meat, and fish [18].

The primary sources of plant proteins in the human diet are legumes (e.g., soya beans, peas, lentils, mung beans, black beans, and chickpeas), cereals (e.g., wheat, maize, and rice), oilseeds (e.g., rapeseed and sunflower), nuts, and seeds [19]. With advances in biotechnology, microbial protein production has gained increasing attention in both academia and industry. First, microorganisms exhibit high protein content. For instance, *Spirulina* has a crude protein content of approximately 70%, whereas *Chlorella* has a protein content of up to 60% and contains all the essential amino acids [20]. Second, microorganisms can synthesise proteins that are absent in higher animals and plants, such as phycobiliproteins (e.g., phycocyanin, phycoerythrin, phycoerythrocyanin, and allophycocyanin), which can reduce oxidative stress by neutralising reactive oxygen species, thereby exhibiting strong antioxidant properties, antibacterial, anticancer, and ultraviolet protection bioactivities [9,21]. Microalgae also synthesise selenoproteins (e.g., thioredoxin reductases; methionine sulfoxide reductases; and selenoproteins W, U, and T) contained in the human body with the significant effect of cancer prevention [22]. Furthermore, microbial proteins can be efficiently produced in closed, intensive bioreactor systems that require little arable land and freshwater and do not require pesticides or antibiotics. Therefore, such proteins can be produced through urban farming on marginal land and in industrialised metropolitan areas [20]. Microorganisms can be used as chassis cells to produce edible vaccines, antibodies, human milk proteins, ovalbumin, casein, lactoferrin, therapeutic proteins, and bioactive peptides [23,24,25]. It is estimated that 800 million vegetarians worldwide will soon gain access to high-quality proteins derived from microbes.

### 2.2. Functional Polysaccharides and Oligosaccharides

Sugars are the most important source of energy for humans, and functional polysaccharides and oligosaccharides are the two types of functional sugars. Functional polysaccharides are non-starch polysaccharides that regulate the physiological functions of the human body. They are categorised into two groups: dietary fibres and active polysaccharides. Plant cell walls contain non-starch polysaccharides such as cellulose, hemicellulose, lignin, and pectin, which are commonly present in celery and citrus fruits [26]. Additionally, high-quality dietary fibres include resistant starch found in microalgal pyrenoids, as well as gum, pectin, and mucilage. Active polysaccharides include animal polysaccharides (chitosan), plant polysaccharides (e.g., tea polysaccharides, *Ginseng* polysaccharides, *Astragalus* polysaccharides, and *Lycium barbarum* polysaccharides), and microbial polysaccharides (e.g., fungal polysaccharides; cellular polysaccharides; and *Euglena gracilis* polysaccharides, also known as β-1,3-glucan or paramylon). Functional polysaccharides can serve as prebiotics in humans. They participate in multiple beneficial biochemical processes, including the synthesis of vital vitamins, activation of the immune system, and fermentation of carbohydrates into short-chain fatty acids, by regulating the activity of microorganisms in the gastrointestinal tract. These polysaccharides demonstrate great efficacy in lowering cholesterol and blood sugar levels, have antioxidant properties, boost immunity, and enhance intestinal function [27,28,29].

Several functional oligosaccharides can be synthesised using microorganisms. For instance, allulose, a new low-calorie sweetener, potentially exerts anti-diabetic and anti-obesity effects and is thus an ideal substitute for sucrose [30]. Other oligosaccharides, including lacto-*N*-neotetraose [31], 2′-fucosyllactose, and 3-fucosyllactose, play crucial roles in regulating immunity, assisting brain development, and regulating intestinal flora. They are commonly found in baby formulas and are advantageous for the growth and development of infants and young children [32].

### 2.3. Polyunsaturated Fatty Acids

Polyunsaturated fatty acids (PUFA) are essential in humans. They are typically categorised into two categories: omega-6 fatty acids (linoleic acid [LA], arachidonic acid [AA]) and omega-3 fatty acids (α-linolenic acid [ALA], γ-linolenic acid [GLA], stearidonic acid [SDA], docosapentaenoic acid [DPA], docosahexaenoic acid [DHA], and eicosapentaenoic acid [EPA]). Omega-6 fatty acids found in soybean, safflower, sunflower, walnut, and corn oils reduce serum low-density lipoprotein cholesterol and triglyceride levels and increase low-density lipoprotein cholesterol (HDL-C) levels, resulting in a significant overall decrease in the total cholesterol/HDL-C ratio. Among the various omega-3 fatty acids, EPA and DHA have garnered considerable attention and are in high demand because of their prominent functional activities [33]. Both EPA and DHA exert key influences on the development and function of the brain and nervous system and are capable of lowering cholesterol levels, reducing platelet aggregation, and decreasing the risk of heart disease and stroke [34]. Fish, such as tuna, are the main sources of EPA and DHA in the human diet. However, fish oils are unsuitable for vegetarians because of their fishy odour and gradually declining fish populations [35]. Therefore, the search for new sustainable sources of fatty acids has become extremely important. Microalgae are considered an ideal alternative source [36]. In particular, *Schizochytrium* sp., *Ulkenia amoeboidea*, and *Crypthecodinium cohnii* have been approved as sources of DHA supplements in infant formulas [34].

### 2.4. Vitamins and Their Analogues

Vitamins are essential nutrients that the body either cannot produce on its own or produces in insufficient amounts. Therefore, they must be obtained daily through food. They have numerous benefits, such as alleviating fatigue, reducing depression, protecting the skin and cardiovascular system, and preventing the development of heart disease, diabetes, Alzheimer’s disease, and cancer [37,38]. However, many vitamins are heat-sensitive, denatured, and inactivated during high-temperature treatment, resulting in insufficient intake, especially vitamin C, vitamin E, vitamin B1, and vitamin B2. Therefore, vitamin deficiencies are common, which is a major cause of hidden hunger in humans. Fruits and vegetables are the primary dietary sources of vitamins in humans. However, microalgae are also rich in vitamins required by the human body, such as vitamins B1 (thiamine), B2 (riboflavin), B3 (niacin), B5 (pantothenic acid), B6 (pyridoxine), B7 (biotin), B9 (folate), B12 (cobalamin), C, D2, E, and K. Additionally, the content of many of these vitamins is much higher in microalgae than in conventional foods, making microalgae a natural source of high-quality vitamins [39,40,41,42]. For instance, β-carotene (converted to vitamin A in humans) synthesised by *Dunaliella salina* under suitable conditions can reach levels more than 10% of the dry weight. Astaxanthin (a carotenoid with antioxidant activity 200 times higher than that of tea polyphenols and 60 times higher than that of coenzyme Q10) produced by *Haematococcus pluvialis* may reach up to 7% of its dry weight [41,43].

### 2.5. Minerals and Trace Elements

Minerals are essential components and functionally active factors in the human body. They participate in various metabolic processes as activators, cofactors, and structural components of various enzymes, and play an indispensable role in maintaining acid-base balance and osmotic pressure stability. Mineral deficiency can lead to hidden nutritional problems. Twenty essential mineral elements support vital human activities, and 16 basic elements are required for plant growth. Na, I, Se, and Co are the four mineral elements that differ between humans and plants. Supplementation with Na, Co, and I can be achieved through inorganic routes; however, the effective and safe supplementation of Se remains a major challenge. This is mainly attributed to the fact that inorganic Se is highly toxic in nature and has a narrow range for safe consumption. Furthermore, Se is not an essential trace element for the growth and development of higher plants. Therefore, it is difficult to achieve accurate and efficient Se supplementation by relying solely on traditional intake of grains, vegetables, and fruits. Microalgae are rich in mineral elements, such as Cu, Fe, Zn, Se, Mu, P, and As, and possess the ability to synthesise selenoproteins [44,45]. For instance, genes encoding 59 selenoproteins have been identified in the selenoproteome of *Aureococcus anophagefferens* [46]. *Chlamydomonas reinhardtii* possesses at least 10 selenoproteins [47], and the *Ostreococcus lucimarinus* genome contains 20 selenocysteine-encoding genes [48]. Thus, the conversion of inorganic Se into selenoprotein-based organic Se through edible microalgae is an effective approach for the utilisation of Se [49], which can potentially serve as a functional food supplement to overcome the problem of inadequate daily Se intake.

### 2.6. Carotenoids

Carotenoids are fat-soluble pigments comprising eight isoprene (C5) units. The conjugated double-bond structure of these molecules confers antioxidant properties on natural pigments [50]. More than 500 different carotenoids have been identified, including astaxanthin, fucoxanthin, β-carotene, lutein, lycopene, canthaxanthin, zeaxanthin, and neoxanthin [51]. These carotenoids are found in high concentrations in plants such as tomatoes, carrots, and corn. Yeasts are also high-quality model organisms for carotenoid synthesis. For carotenoid biosynthesis, acetyl-coenzyme A (Ac-CoA) is condensed into five carbon molecules, namely IPP (iso pentenyl diphosphate) and DMAPP (dimethylallyl diphosphate). Through a downstream carotenoid biosynthetic pathway, IPP and DMAPP are sequentially condensed to yield carotenoid molecules, such as lycopene, β-carotene, astaxanthin, and zeaxanthin, among others [52]. Notably, the antioxidant capacity of naturally extracted carotenoids is superior to that of chemically synthesised products. In particular, the antioxidant activity of astaxanthin is 500 times that of vitamin E and 6000 times that of vitamin C. Given their powerful antioxidant properties, carotenoids have the potential for a wide range of applications in medicine and healthcare, such as adjuvant therapy for Parkinson’s disease, the treatment of metabolic syndrome, maintenance of oral health, sun protection for the skin, and the alleviation of hyperglycaemia [51,53,54,55].

## 3. Strategies to Improve the Nutrient Density of Diets

Nutritional density refers to the concentration of nutrients in food; the greater the nutrient density, the higher the nutrient richness of the food. The nutrient density of diet is closely linked to health status. Hidden hunger is an insidious form of malnutrition caused by the lack of essential vitamins and minerals required by the human body. To address hidden hunger, several strategies have been adopted to optimise nutrient intake. These include direct nutrient supplementation, food fortification, the promotion of diet diversification, biofortification, and multidimensional methods, such as the use of toxicity attenuation and synergy. The latter two approaches can cultivate food materials rich in specific health-promoting ingredients, which are subsequently used as raw materials for in-depth processing to develop functional foods with health benefits, thus improving the nutrient density of diets [56,57].

### 3.1. Biofortification

Biofortification techniques can be implemented through an agronomic pathway, whereby the concentration of mineral elements in exogenous nutrient solutions is adjusted to promote the accumulation of target minerals in the biological host. For instance, while the use of sodium selenite tablets as a selenium supplement poses safety concerns, the primary organic sources of selenium in the daily diet are fish, shellfish, and animals. However, these sources frequently include potentially hazardous inorganic forms of Se [58,59]. Recent studies have revealed that because of their unique metabolic mechanisms, microalgae can efficiently convert inorganic Se into organic Se, which has low toxicity and high bioavailability, with a conversion efficiency of more than 80% [49,60]. This discovery provides a novel natural biological solution for the safe and effective fortification of foods with Se.

Genetic biofortification strategies rely on genetic breeding techniques aimed at cultivating or optimising new crop varieties with enhanced mineral element accumulation capacities. Modern biotechnological techniques, such as genetic engineering, synthetic biology, and gene editing, provide key support for food fortification. In addition to the efficient biosynthesis of key nutrients, such as proteins [61], polysaccharides [32,62], and PUFAs [39,63], these technologies greatly enhance the nutritional density and functional qualities of foods and create new paths for nutritional improvement and health promotion. Mapelli et al. adopted a metabolic engineering approach for the expression of heterologous selenocysteine methyltransferase in *Saccharomyces cerevisiae*, resulting in an approximately 24-fold increase in selenocysteine content [64]. Li et al. used CRISPR-Cas9-mediated genome editing to knock down five alleles in tomatoes, including *lycopene ε-cyclase*, *lycopene β-cyclase1*, and *lycopene β-cyclase 2*. This promoted the accumulation of lycopene, while inhibiting the conversion of lycopene to β- and α-carotene, leading to an approximately five-fold increase in lycopene content [65]. Similarly, Li et al. knocked down the *7-dehydrocholesterol reductase* allele using CRISPR-Cas9 genome editing to induce the accumulation of 7-dehydrocholesterol without affecting the growth, development, or yield of tomato plants [66]. More notably, Waltz reported the use of CRISPR-Cas9 gene editing to produce tomatoes with a γ-aminobutyric acid-enriched content higher than mulberry leaves and 4–5 times higher than that of conventional tomatoes. These tomatoes have been approved for marketing [67].

### 3.2. Plant Biotechnological Detoxification and Synergism of Plants

Enhancing the nutritional level of crops through nutrient fortification and eliminating potential antinutritional metabolites is essential for the production of functional food ingredients. Phytotoxins are toxic secondary metabolites produced by plants as a defence against herbivores and pathogens. These compounds include cyanogenic glycosides, glucosinolates, alkaloids, and terpenoids. For instance, rapeseed and cassava leaves (containing cyanogenic glycosides), potato tubers and fruits (containing solanine), and seeds of leguminous crops (containing β-diaminopropionic acid [β-ODAP]) possess the potential for crop/organ development but contain natural toxins. Biotechnological strategies for the removal of antinutritional metabolites include (1) knocking out key catalytic enzymes to inhibit the biosynthesis of antinutrients, (2) targeting upstream transcription factors, (3) disrupting the transport or storage of antinutrients, and (4) converting antinutrients into non-toxic substances [68]. For instance, Gomez et al. knocked out CYP79D1 and *CYP79D2* in cassava using CRISPR-Cas9 genome editing, which significantly reduced the synthesis of cyanogenic glycosides [69]. Kumar et al. utilised genetic engineering techniques to introduce a fungal oxalate decarboxylase gene into grass pea for the degradation of oxalate, thereby reducing the β-ODAP concentration in grass pea seeds by 73% [70]. The application of these strategies will help improve the nutritional value of crops. Thus, these strategies have immense potential for use in functional agriculture.

## 4. Industrial Production of Functional Foods

**Figure 2:**
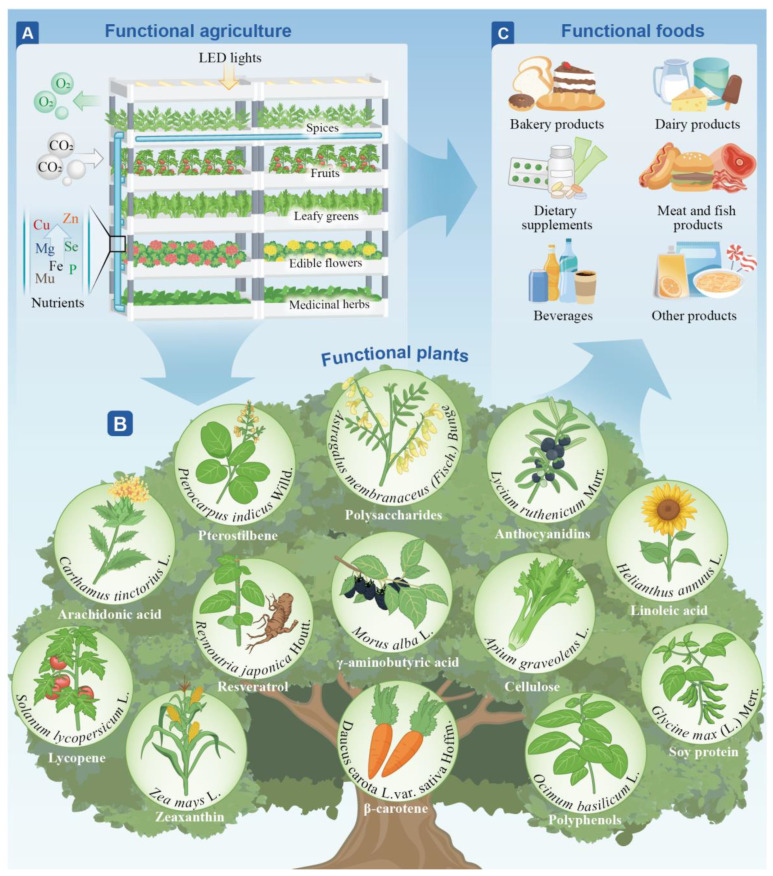
An industrial chain that shifts from functional agriculture (**A**) to functional plants (**B**) and, eventually, to functional food (**C**). This pathway, which is based on functional agriculture, involves the selective utilisation of plants as biological platforms for nutrient fortification to cultivate functional products rich in specific health-promoting ingredients. These ingredients are subsequently used as raw materials for in-depth processing to develop functional foods with health benefits.

A significant trend in the agriculture and food industries is the technological advancement of agriculture and the development of an industrial chain that shifts from functional agriculture to functional plants, and eventually, to functional food. This process, which is based on functional agriculture, involves the selective utilisation of plants or microorganisms as biological platforms for nutrient fortification to cultivate functional products rich in specific health-promoting ingredients. Subsequently, these ingredients are used as raw materials for in-depth processing to develop functional foods with health benefits (Figure 2). Therefore, the establishment of efficient plant and cell factory production systems is a key strategy for achieving the efficient utilisation of resources and increasing the production yield and efficiency of functional foods.

### 4.1. Plant Factories

Plant factories are efficient agri-food systems that enable year-round planned production of crops in an indoor three-dimensional space through high-precision environmental control. Computers are used to accurately control the temperature, humidity, light, carbon dioxide concentration, nutrient solution, and other environmental conditions required for plant growth and development to obtain high-quality and high-yield agricultural products. Compared to traditional agriculture, plant factories offer multiple advantages, including reduced land requirements, a short production cycle, high production efficiency, lack of geographical limitations, high product safety, standardised quality, and the capability for nutritional customisation [71,72]. Plant factories have been established worldwide and mainly produce functional products such as leafy greens, fruits, medicinal plants, spices, and edible flowers. The market size was approximately USD 5.1 billion in 2023 and is expected to increase to USD 15.3 billion by 2030, with a production output of more than 600,000 tonnes [73]. Hu et al. reported that the use of a plant factory to grow rice led to the shortening of the growth cycle from 120 d in a conventional paddy field to 60 d [74,75]. Another study by Liao et al. indicated that the efficiency of food production in plant factories was 400 times higher than that in traditional soil-based agriculture [75]. Miyagi et al. showed that the biomass and amino acid levels of head lettuce can be significantly increased by optimising the amount of red light, CO_2_, and nutrient formulation [76]. Similarly, medicinal plants, such as basil, exhibited significantly higher antioxidant activity and increased protein and soluble sugar content when subjected to UV light irradiation in a plant factory [77]. Three types of cultivation modes are generally adopted in plant factories: hydroponics, aeroponics, and aquaponics, all of which require less water and fertiliser and generate lower levels of pesticide residues than traditional methods. Among these modes, aquaponics exhibits the highest water utilisation efficiency, safety, and controllability. However, its investment cost is also the highest, which has led to the widespread adoption of the other two modes. For example, Vertical Harvests uses hydroponics to supply the market with approximately 100,000 pounds of microgreen, lettuce, and tomatoes annually, and the adoption of aeroponics by AeroFarms has led to a 95% reduction in water usage and pesticide residue levels [73]. With limited land and controlled environments to produce safer and nutritious food, plant factories have become indispensable for eradicating hunger and ensuring food security and human health.

However, high energy demand, capital costs, and limitations of crop variety are major challenges in achieving the triple pillars (planet, people, and profit) of sustainability. Future research should focus on the utilisation of renewable energy sources (e.g., geothermal energy, solar energy, wind energy, and hydropower) for heating, ventilation, air conditioning, and dehumidification and achieve precise control with energy-efficient lighting technologies and digital twin platforms. This makes it possible to lower the energy and labour expenses associated with environmental control. Research should also be conducted on plant factory cultivation technologies for high-value-added plants, such as medicinal plants, health-promoting plants, and rare vegetables, to increase the variety of crops cultivated in plant factories [78].

### 4.2. Microbial Cell Factories

**Figure 3:**
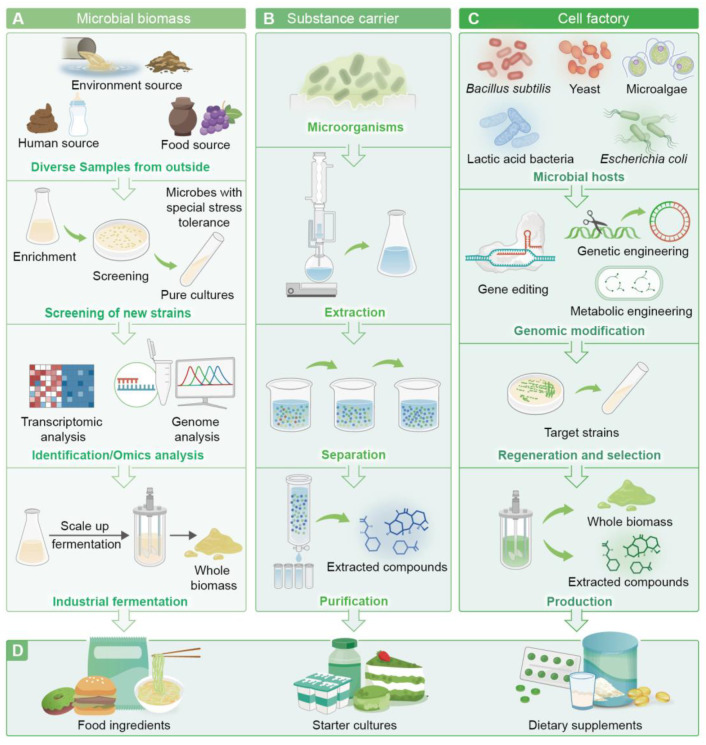
Pathways for the industrialised production of functional foods. There are three main pathways through which microorganisms are applied in food production. Application of microbial biomass (**A**): Microbial biomass is obtained from microorganisms sourced from food, humans, and the environment through screening, identification, and fermentation. Application of substance carriers (**B**): Microorganisms can synthesise various high-value bioactive ingredients, which are then extracted, separated, and purified to obtain pure substances. Application of cell factory (**C**): Cell factory refers to the use of microorganisms as production hosts to achieve the industrialisation of target products. Applications in food (**D**): The microbial biomass or extracted compounds obtained from the three above-mentioned methods are used as food ingredients, starter cultures, or dietary supplements for food production.

Microorganisms play a pivotal role in functional foods and are primarily used in three ways: (1) microbial biomass applications, (2) microbial substance carriers, and (3) microbial cell factories (Figure 3). The term ‘cell factory’ first appeared in the 1970s and refers to the use of microorganisms as production hosts in biotechnology to produce microbial enzymes and industrially important biochemicals by metabolism. However, because of the complexity of microbial cell networks, only a few species (e.g., *Escherichia coli*, *Bacillus subtilis*, *Lactic acid bacteria*, *Saccharomyces cerevisiae*, and microalgae) are capable of serving as high-quality microbial hosts that utilise inexpensive carbon sources as substrates, maintain a stable state during the fermentation process, possess a clear genetic background, are non-pathogenic, and do not contain exotoxins or endotoxins. Cell factories have the advantages of fast growth rate, strong metabolic capacity, relatively simple culture conditions, and capability for large-scale production [79]. The optimisation of expression systems and culture conditions using mature tools and strategies in metabolic engineering, genetic engineering, gene editing, and high-cell-density culture will enable the efficient and economical production of bioactive substances of plant and animal origins [80]. Microorganisms are widely used in the food industry.

Kang et al. improved the efficiency of carotenoid synthesis in engineered *E. coli* by 5.7-fold and increased the yield of lycopene synthesis in *S. cerevisiae* to 2300 mg/L using a modular enzyme assembly [81]. The use of different wine yeasts and enzymatic preparations was responsible for the extraction of resveratrol during fermentation [75,82]. Yang et al. constructed recombinant strains of *E. subtilis* using metabolic engineering, which enabled an increase in the vitamin K2 concentration to 281.4 ± 5.0 mg/L in a 5 L fermenter [42]. Lactic acid bacteria have been used in the commercial production of a wide range of food additives, including sweeteners (e.g., mannitol and xylitol), flavouring agents (e.g., acetaldehyde and diacetyl), and vitamins. Pathways for the synthesis of polyphenols, such as resveratrol (primarily derived from *Reynoutria japonica*), pterostilbene (primarily derived from *Pterocarpus santalinus*), and anthocyanidins (primarily derived from black wolfberry), have been successfully constructed using bacteria [83], providing a basis for the mass production of high-value polyphenols. Microorganisms are ideal alternatives for animal-based foods. Using single-cell proteins from the filamentous fungus *Fusarium venenatum*, Quorn Foods successfully created meat replacements that are now consumed in 17 countries [84,85]. Soy leghaemoglobin, synthesised in *Pichia pastoris*, can serve as a sustainable source of flavour and aroma, with low allergenicity in plant-based meats [86]. Other studies have successfully expressed human milk oligosaccharides, human milk fats, casein, and whey proteins in *S. cerevisiae*, *E. coli*, and *B. subtilis* [9,32,63,87,88], which can be consumed by vegetarians.

It is particularly noteworthy that microalgae have survived five mass extinctions, and thus offer unparalleled advantages over other microorganisms. In addition to being highly diverse (200,000–800,000 species) and having an exceptionally high nutrient content, microalgae possess the combined advantages of animals, plants, and microorganisms, are wholly edible, and enable efficient energy utilisation. These features make them attractive alternatives to the derivation of natural animal and plant products by extraction from animals and plants or chemical synthesis. Currently, microalgae are produced and processed in more than 30 countries. In particular, *Spirulina* is recognised by the World Health Organisation as one of the major ‘superfoods’, and this has prompted a new wave of applications of microalgae as functional food ingredients. Credence Research reported that the global algae products market was valued at USD 4.5 billion in 2021 and is projected to reach a value of more than USD 6.3 billion by 2028 [89].

Advances in science and technology have contributed to the growth of the microalgal industry. For example, Rathod et al. heterologously expressed the *CrtYB* (*phytoene-β-carotene synthase*) gene from *Xanthophyllomyces dendrorhous* in *Chlamydomonas reinhardtii* and found that light induction resulted in 72% and 83% increases in β-carotene and lutein yields, respectively [90]. Jeon et al. employed the CRISPR-Cas9 system to generate chlorophyll synthase loss-of-function mutants that exhibited an increase in phycoerythrin content to 37.07 mg/L [91]. Microalgae are widely used in food production (Table 1). Despite these promising results, the commercialisation of microalgae faces technical bottlenecks in scaling up standardised cultures, low-cost dehydration, and functional component extraction. Once these technical bottlenecks are addressed, microalgal cell factories, which serve as potential novel food production methods, will possess a higher market value and contribute more to the sustainability of food production.

## 5. Challenges Facing Functional Food Development

### 5.1. High Cost

**Figure 4:**
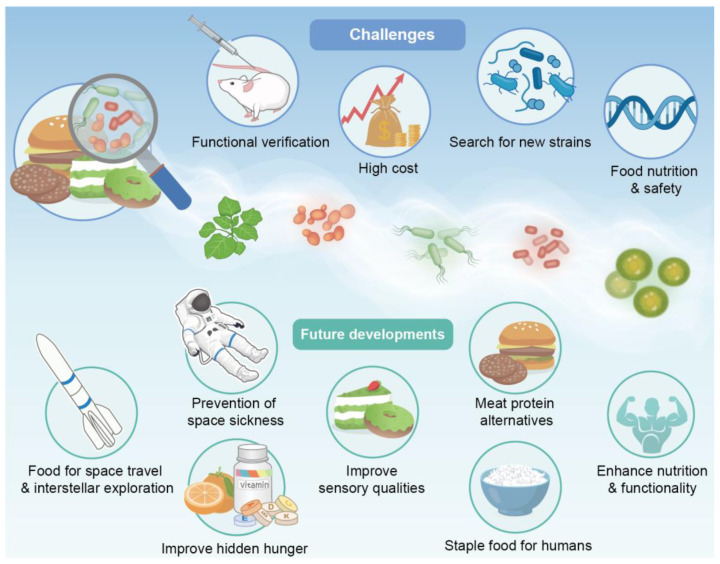
Challenges and future development of functional foods. Functional foods face numerous challenges, including their high cost, the search for new strains, functional verification, and ensuring food nutrition and safety. However, they also have significant potential for future development, such as serving as food for space travel and interstellar exploration, preventing space sickness, and potentially becoming staple foods for humans, among other applications.

Certain challenges must be overcome to fully utilise plants and microorganisms in food production (Figure 4), one of which is high cost. First, large-scale culture of microorganisms is extremely expensive [112]. This is partially due to the high construction and operational costs of photobioreactors [113]. Harvesting costs, which make up 20–30% of the total production cost, are caused by the small size of microbial cells and the incompatibility of chemical harvesting techniques with food applications [114]. To maintain the activity of functional substances, measures such as cold-chain transport, microencapsulation techniques [115], and freeze-drying technology [116] are usually adopted, which increases the costs of food technology and transport. The purity of the natural bioactive ingredients required for food products must also be higher than that required for feed applications, which increases the cost of processing [117]. Finally, the commercial use of microbiological foods remains heavily regulated, and the long and complex safety approval process poses a major challenge [118].

### 5.2. Search for New Strains

The search for new strains through the screening and modification of functional plants and microorganisms in food products is challenging. Screening and domestication combined with random mutagenesis or genetic engineering is an effective strategy for obtaining desired strains. However, an imbalance between yield and quality remains [16]. For instance, certain microorganisms possess high biomass and low lipid content, and vice versa. Therefore, for specific biologics, the search for strains with both high growth rates and high concentrations of bioactive ingredients is a difficult task in bioprospecting [119]. In addition, certain strains may produce toxins, which reduces their applicability and increases the difficulty of screening [114].

### 5.3. Food Nutrition and Safety

Plants and microorganisms are rich in bioactive ingredients but may also contain toxic or potentially toxic substances that are hazardous to human health (e.g., solanine in potato tubers and fruits, cyanogenic glycosides in cassava leaves, β-ODAP in seeds of leguminous crops, and nucleic acids in microorganisms). These substances must be eliminated during food processing [120]. Another significant obstacle faced by the functional food industry is maintaining the integrity and activity of functional ingredients during processing and storage [121]. Natural bioactive ingredients may interact or react with other ingredients, resulting in insolubility, oxidation, precipitation, or degradation [122]. The combined use of functional ingredients may aid in the maintenance of the stability of food products but may also affect their digestion and metabolism and reduce their bioavailability after consumption. For example, milk exerts a negative effect on the metabolic pathways of flavonoids; therefore, it should not be used with combination [123]. Therefore, extensive research is required to ensure food security, accomplish the goal of ending hunger, and encourage the production of high-quality nutritious foods for many people [16].

### 5.4. Functional Verification

Although the functional food industry is growing, relevant laws and regulations, including the verification of food product functionality, are lacking. This has led to the unclear functionality of certain foods. Food digestion and absorption involve multistage processing in the oral cavity, oesophagus, stomach, and intestine. Mechanical chewing in the oral cavity, the highly acidic environment of the stomach, and decomposition of the intestinal flora may lead to the inactivation of functional ingredients, resulting in their inability to perform their functions [124]. Therefore, the lack of functional verification is an important issue that must be urgently addressed.

## 6. Conclusions and Outlook

With rapid population growth and challenges related to land, water resources, and the environment, malnutrition and hidden hunger are becoming increasingly prominent, fulling a strong consumer interest in healthy eating habits. Functional foods have the advantage of “replacing medicine with food” and have become the best choice.

The efficacy of functional foods comes from the bioactive substances contained in the raw materials, through biotechnological, agronomic, and breeding techniques, the quality and quantity of bioactive ingredients can be improved, and at the same time, genetic engineering, gene editing and genetic engineering and other biotechnology can reduce the content of harmful substances in raw materials, and further ensure the efficacy of functional foods, which is also a manifestation of the vigorous development of the bioeconomy. The application of plant factories and cell factories has laid the foundation for the industrial production of functional foods to provide safe, green, and nutritious raw materials through precise environmental control, and promote the vigorous development of the functional food industry. However, this article still has the following gaps and limitations: On the one hand, its content regarding the industrial production of functional foods is not comprehensive; for example, non-thermal processing methods and active substance extraction methods are not mentioned. On the other hand, the article contains a wide range of content, and each part of the content lacks depth and focus, which should be avoided in future writing.

In the future, although there are still some challenges to be overcome, functional foods may play a key role in other areas, including, but not limited to, meat protein alternatives, stable food for humans, and disease prevention, and build a solid foundation for achieving the 17 Sustainable Development Goals. With greater exploration of the universe, it is expected that functional foods may also serve as crucial support for space exploration by providing astronauts with the necessary nutrition and energy resources.

## Figures and Tables

**Table 1:** Microalgae incorporation in food products.

Microalgae	Ingredients	Products/Applications	Function/Benefits	References
*Chlorella vulgaris*	Biomass	Wheat bread dough	Changed the colour, texture, and antioxidant capacity of food.	[92]
*Spirulina maxima*	Biomass	Vegan biscuits	Dramatically increased the protein, iron and PUFA content of biscuits without altering sensory acceptance.	[93]
*Dunaliella salina* and *Chlorella vulgaris*	Biomass	Fresh green smoothies	Increased the sensorial properties, microbiological quality and phenolic contents of food.	[94]
*Spirulina platensis*	Biomass	Chocolate shakes	Increased the content of protein and fibre, extend the shelf life, and the product has good sensory acceptance.	[95]
*Spirulina platensis*	Biomass	Cassava doughnuts	Not only improved the nutritional quality of the doughnut, but also improves the acceptance.	[96]
*Haematococcus pluvialis*	Biomass	Cookies	Improved bioactive composition of cookies.	[97]
*Arthrospira* sp.	Biomass	Extruded snacks	Made food with high nutritional value and sensory acceptance	[98]
*Spirulina platensis*	Biomass	Cheese	The antioxidant capacity of cheese has been increased by nearly 10 times.	[99]
*Isochrysis galbana* and *Diacronema vlkianum*	Biomass	Pasta	Addition of omega-3 polyunsaturated fatty acids.	[100]
*Spirulina platensis*	B Complex vitamins	Health supplement	Added nutritional properties (Help the body to convert food in energy).	[39]
*Nannochloropsis oceanica*	Vitamin D3	Health supplement	Added nutritional properties (Development and maintaining of skeleton; regulation of blood pression; cardiovascular protection).	[101]
*Chlorella ellipsoidea*	Peptides	Functional food ingredients	Enhanced antioxidant and antihypertensive properties of food.	[102]
*Spirulina platensis*	Protein	Animal product alternatives/Meat analogues	Simulated the structure and texture of meat analogues.	[84]
*Spirulina platensis*	Phycocyanin	yoghurt	Maintained the colour stability of ice cream for 182 days and increased its antioxidant activity.	[103]
*Haematococcus pluvialis*	Astaxanthin	Nutritional supplement	Imparted colour to food as well as strong antioxidant properties	[104]
*Thraustochytriidae* sp.	Exopolysaccha-rides	Food additives	Increased food consistency, anti-proliferative and immunomodulatory effects.	[105]
*Euglena gracilis*	β-1,3-glucan	Nutritional supplement	An active immunostimulant and blood lipids reductor.	[45]
*Dunaliella salina*	β-carotene	Juice, can	Imparted bright colours to food and improved antioxidant properties.	[106]
*Porphyridium purpureum*	Phycoerythrin	Bio-colorant	Gave bright colour and functionality to food.	[107]
*Schizochytrium* sp.	EPA and DHA	Spread, dressing	It is important for the maintenance of membrane fluidity and the development of the brain and retina.	[34]
*Chlamydomonas reinhardtii*	Lutein, zeaxanthin	Beverage, functional food and supplement	Used as a pigment while imparting antioxidant properties to food.	[106]
*Arthrospira platensis*	Gamma-linolenic acid	Oil	Mediated immune process; prevented several chronic inflammatory diseases and cancers;	[108]
*Spirulina*, *Chlorella* spp.	Ascorbic acid	Doughnuts, spaghetti, Biscuits	Prevention of oxidation of biomolecules	[109]
*Euglena gracilli*, *Dunaliella salina*	Tocopherol	Vitamin supplement	Prevented the oxidation of saturated fatty acids	[110]
*Scenedesmus* sp., *Phaeodactylum tricornutum*	Phenolic compounds	Functional food	Antioxidant; antiviral; antiproliferative; regulation of macronutrient digestion	[111]
*Desmodesmus intermedius*, *Dictyosphaerium*	Calcium	Food supplement	Structural; enzyme cofactor; blood clotting; muscle and nerve function	[44]
*Chlorococcum humicola*, *Coccomyxa simplex*	Cooper	Food supplement	Essential in metabolic processes; haemoglobin and enzyme formation	[44]
*Spirulina*, *Thalassiosira* sp.	Zinc	Dietary supplement	Managed stress; protein synthesis; immune and enzyme systems	[16]
*Arthrospira platensis*	Selenium	Dietary supplement	Necessary for metabolic processes	[45]
*Aureococcus anophagefferens*	Selenoproteins	Dietary supplement	Avoided Keshan disease, cardiovascular disease and myocardial infarction	[46]

## Data Availability

No new data were created or analyzed in this study. Data sharing is not applicable to this article.
